# Coordinated Hippo, IFN and NF-κB pathway activation in kidney rejection

**DOI:** 10.1515/med-2026-1516

**Published:** 2026-09-08

**Authors:** Eda Balkan, Murat Kızılkaya, Mahmut Aktürk, Elif Demirci, Necip Altundaş

**Affiliations:** Department of Medical Biology, Tissue Typing and Transplant Immunology Laboratory, Faculty of Medicine, Atatürk University, Erzurum, Türkiye; Department of Medical Biology, Faculty of Medicine, Ağrı İbrahim Çeçen University, Ağrı, Türkiye; Department of Pathology, Faculty of Medicine, Atatürk University, Erzurum, Türkiye; Department of General Surgery, Faculty of Medicine, Atatürk University, Erzurum, Türkiye

**Keywords:** calcineurin inhibitor, *ISG15*, non-invasive biomarker, renal transplant rejection, *YAP1/TEAD1*

## Abstract

**Objectives:**

Renal allograft rejection diagnosis requires invasive biopsy. We assessed whether peripheral blood mRNA of 14 Hippo, interferon (IFN) and NF-κB pathway genes and 11 serum biomarkers could distinguish acute rejection from stable graft function.

**Methods:**

Three groups: biopsy-confirmed acute rejection (n=20), stable graft (n=25), healthy controls (n=30). PBMC mRNA was quantified by qRT-PCR; serum biomarkers by ELISA and spectrophotometry. Statistical analyses used Mann–Whitney *U* with Benjamini–Hochberg correction; ROC analyses used bootstrap 95 % CIs and 5-fold cross-validation. GEO datasets GSE51675 and GSE145408 provided directional cross-validation.

**Results:**

Thirteen of 14 genes were upregulated in rejection vs. controls (pFDR<0.01); *TEAD1* (log_2_FC=+2.44) and *TRAF6* (+2.40) led. *ISG15* was downregulated in both transplant groups, consistent with calcineurin inhibitor-mediated suppression, while serum free ISG15 protein was unchanged in stable graft but markedly lower in rejection than in both controls and stable graft (pFDR<0.001 and 0.002), discriminating rejection from stable graft in a way the mRNA signal did not. Only *TEAD1* (Cohen’s *d*=1.40) and *YAP1* (*d*=1.17) discriminated rejection from stable graft with adequate power. *TEAD1* mRNA (AUC=0.849) and serum leptin (AUC=0.922) were the strongest single markers; their combination yielded cross-validated AUC=0.990.

**Conclusions:**

PBMC *YAP1/TEAD1* with serum leptin are promising non-invasive biomarkers for acute renal rejection. *ISG15* downregulation is more consistent with calcineurin inhibitor-mediated suppression than with generalized immune impairment, although this interpretation requires direct mechanistic confirmation; notably, serum free ISG15 protein discriminated rejection from stable graft even though PBMC ISG15 mRNA did not, suggesting the protein-level measurement warrants further evaluation as a candidate marker. Prospective multicentre validation is required.

## Introduction

Renal transplantation is the optimal treatment for end-stage renal disease, but acute rejection remains a leading cause of graft loss [1], 2]. The diagnostic gold standard – transplant kidney biopsy – is invasive and cannot be repeated at will. This has driven interest in peripheral blood markers that can flag rejection without tissue sampling [3].

The Hippo pathway effectors YAP1 and TEAD1 regulate cell proliferation and tissue homeostasis through downstream targets *CTGF* (CCN2), *CYR61* (CCN1) and *ANKRD1*; *LATS1* restrains this axis via YAP1 phosphorylation [4]. In kidney disease, YAP/TAZ drives ischaemia-reperfusion injury, tubular proliferation and fibrosis [5], 6]. *CTGF*/CCN2 is a classical renal fibrosis marker [7]. YAP1 is not restricted to parenchymal cells: it promotes Th17 T cell differentiation and survival [8] and modulates macrophage inflammatory activation [9], making it directly relevant to PBMC-based profiling.

In the NF-κB pathway, *MYD88*, *TRAF6*, *NEMO*, *HOIP* and *HOIL-1* – the catalytic and regulatory LUBAC subunits – amplify TLR-driven inflammatory programmes [10]. Interferon (IFN) pathway genes *IFNB1*, *IFNGR1* and the downstream ISGylation effector *ISG15* are involved in transplant pathology [11], but their expression depends partly on the drug regimen. Calcineurin inhibitors (CNI) block NFAT-mediated IFN-γ transcription in T cells [12], reducing downstream IFN-stimulated gene (ISG) expression including *ISG15* [13]. Coordinated activation of all three axes has been described in rejection [14].

Most published work compares rejection with healthy controls and stops there. That misses the comparison a clinician actually needs: rejection vs. a stable transplant. We profiled 14 genes and 11 serum biomarkers across three groups – rejection, stable graft and controls – integrated Banff histopathological scoring, and queried two public Gene Expression Omnibus (GEO) datasets for independent directional cross-validation. Our hypothesis was that a subset of Hippo, IFN and NF-κB pathway transcripts in PBMCs, in combination with serum biomarkers, would discriminate biopsy-confirmed acute rejection from stable graft function with diagnostic-grade performance.

## Methods

### Study design and participants

This prospective case-control study was approved by the Institutional Research Ethics Committee of the lead author’s institution (approval number: B.30.2.ATA.0.01.00641/2025; date: 15 March 2024) and conducted in accordance with the Declaration of Helsinki. Three groups were enrolled: (i) Rejection (n=20), biopsy-confirmed acute rejection patients aged ≥18 years; (ii) Stable graft (n=25), recipients with stable eGFR (change <15 %) over ≥6 months post-transplantation; and (iii) Control (n=30), age- and sex-matched healthy individuals without transplantation, inflammatory disease or malignancy. Written informed consent was obtained from all participants prior to enrolment.

### Clinical characteristics

In the rejection group, 12 patients received living-donor and 8 cadaveric transplants. Serum creatinine at biopsy-confirmed rejection was 6.05 mg/dL (median; range 2.65–12.60). HLA mismatch profiles are presented in Supplementary Table S2. Rejection samples, like stable graft samples, were collected more than 6 months post-transplantation, so time-since-transplant is comparable between these two groups and does not confound the rejection-versus-stable comparison. All patients in both the stable graft and rejection groups were maintained on a standard triple immunosuppressive regimen – a calcineurin inhibitor (predominantly tacrolimus, with cyclosporine in a minority of patients) as the backbone agent, combined with mycophenolate mofetil (or an equivalent antiproliferative agent) and corticosteroids, with steroid dosing typically intensified during acute rejection episodes. However, individual-level dosage, trough drug concentrations, and precise steroid dosing were not systematically recorded for all participants in this retrospective cohort and could not be entered as covariates in the statistical analysis; the impact on *ISG15* and TNF-α interpretation is addressed in the Discussion.

### RNA isolation, cDNA synthesis and qRT-PCR

Peripheral blood mononuclear cells (PBMCs) were isolated from heparinised whole blood by Ficoll-Paque (GE Healthcare, Uppsala, Sweden) density-gradient centrifugation. Total RNA was extracted using a column-based kit with on-column DNase I digestion (Qiagen, Hilden, Germany). RNA concentration and purity were assessed by NanoDrop spectrophotometry (Thermo Fisher Scientific, Waltham, MA, USA); samples with A260/280≥1.8 were retained. Complementary DNA was synthesised from 1 µg total RNA using a commercial reverse-transcription kit. Commercially pre-validated Qiagen primer assays were used for all target genes. SYBR Green qRT-PCR was performed in technical duplicate on a Qiagen Rotor-Gene Q thermocycler (Qiagen, Hilden, Germany): 95 °C for 10 min, followed by 40 cycles of 95 °C for 15 s and 60 °C for 30 s, with melt-curve acquisition; no-template (negative) controls were included in every run, and amplification curves showed consistent, on-target Ct timing with no evidence of inefficient or non-specific amplification. As these were commercially pre-validated primer assays, amplification efficiency was not independently re-determined in-house via a dilution-series standard curve; we relied on the manufacturer’s pre-validation of these reference assays, and note in-house efficiency re-verification as a planned addition for future work. *GAPDH* served as the reference gene (stability confirmed: coefficient of variation 3.7–4.8 %; Kruskal–Wallis p=0.776). Multi-gene normalisation with *ACTB*/*HPRT1* is planned in line with MIQE guidelines [15], 16]. Relative expression was calculated by the 2−ΔΔCt method [17], 18].

### Serum biomarker measurements

Interleukin-6 (IL-6), tumour necrosis factor-α (TNF-α), leptin, adiponectin, free ISG15 protein, superoxide dismutase (SOD), catalase, glutathione peroxidase (GPx), paraoxonase-1 (PON-1), malondialdehyde (MDA) and myeloperoxidase (MPO) were all measured by commercial sandwich enzyme-linked immunosorbent assay (ELISA) kits (catalogue numbers: IL-6, YLA0052HU; TNF-α, YLA0041HU; leptin, YLA1318HU; adiponectin, YLA0015HU; free ISG15, YLA0942HU; SOD, YLA0105HU; catalase, YLA0312HU; GPx, YLA0488HU; PON-1, YLA1120HU; MDA, YLA0562HU; MPO, YLA0610HU), according to manufacturer protocols, with standard curves run on each plate. Assay measurement ranges and detection limits were: IL-6, 1.56–100 pg/mL (LOD<0.5 pg/mL); TNF-α, 15.6–1,000 pg/mL (<5 pg/mL); leptin, 0.156–10 ng/mL (<0.06 ng/mL); adiponectin, 0.156–10 μg/mL (<0.05 μg/mL); free ISG15, 0.15–4 ng/mL (<0.051 ng/mL); SOD, 0.5–16 U/mL (<0.21 U/mL); catalase, 1.5–40 U/mL (<0.65 U/mL); GPx, 2–60 U/mL (<0.85 U/mL); PON-1, 0.312–10 ng/mL (<0.12 ng/mL); MDA, 0.5–16 nmol/mL (<0.22 nmol/mL); MPO, 0.156–10 ng/mL (<0.065 ng/mL). Reduced glutathione (GSH) was measured by a validated spectrophotometric reference method. All assays were run in duplicate; intra- and inter-assay coefficients of variation were below 10 %.

### Banff histopathological assessment

Biopsy specimens were scored by two independent nephropathologists according to the Banff 2019 criteria [2], with consensus reached in cases of disagreement. Scores recorded: i (n=18), t (n=17), g (n=8), ptc (n=6), C4d (n=4), ci (n=9), cg (n=8). All Banff correlations are exploratory given the small subgroup sizes.

### Statistical analysis

Normality was tested by Shapiro–Wilk; variance homogeneity by Levene’s test. Three-group comparisons used Kruskal–Wallis; pairwise comparisons used Mann–Whitney *U*; categorical variables used the chi-square test. Multiple-testing control used Benjamini–Hochberg false-discovery-rate correction (pFDR<0.05). Effect sizes are reported as Cohen’s *d *[19]. Receiver operating characteristic (ROC) area under the curve (AUC) was estimated with 2,000-iteration stratified bootstrap 95 % confidence intervals (CIs) and 5-fold cross-validated AUC; cut-points used Youden’s index [20]. Banff associations used Spearman rank correlation. All analyses were performed in Python 3.12 (scikit-learn 1.3, SciPy 1.11, statsmodels 0.14).

No *a priori* sample-size calculation was performed for this exploratory study. A post-hoc power analysis was conducted for the primary endpoint (*TEAD1* mRNA discrimination, rejection vs. stable graft). The observed Cohen’s *d* was 1.40 (pooled SD=1.28); n=20 vs. n=25; two-tailed α=0.05; power=0.997. For *YAP1*, *d*=1.17; power=0.973. Genes with *d*<0.8 were considered underpowered, and null findings for these in the rejection-versus-stable graft comparison are treated as inconclusive rather than negative (Supplementary Table S3).

### External directional validation

Two public GEO datasets were downloaded using GEOparse v2.0.4 and analysed by Mann–Whitney *U* with Benjamini–Hochberg correction. GSE51675 (Agilent GPL6480 microarray) contains PBMC expression data comparing chronic antibody-mediated rejection (CAMR; n=10) with stable graft recipients (n=8) [21], 22]. GSE145408 (Affymetrix GPL13158; CTOT-09 study) contains PBMC expression data from stable kidney transplant recipients randomised to tacrolimus withdrawal (n=18) vs. tacrolimus maintenance (n=18; paired design) [23]. We searched the public GEO repository for PBMC transcriptomic datasets in kidney transplant recipients and were unable to identify a publicly available dataset profiling PBMC gene expression specifically in biopsy-confirmed acute cellular rejection with an appropriate stable-graft comparator; GSE51675 and GSE145408 were the closest available datasets able to speak to the direction of the Hippo/NF-κB/IFN signalling changes reported here. Neither dataset is a direct model of acute cellular rejection – GSE51675 reflects chronic, antibody-mediated pathology, and GSE145408 contains no rejection episodes at all – so the aim was directional cross-validation rather than statistical replication, given the limited sample size and indirect clinical match of these datasets.

## Results

### Demographic characteristics

The three groups were matched for age (control 37.3 ± 5.0; stable 38.4 ± 5.6; rejection 38.9 ± 8.9 years; Kruskal–Wallis p=0.733) and sex (control 15 M/15 F; stable 20 M/5 F; rejection 12 M/8 F; chi-square p=0.070) (Table 1).

**Table 1:** Demographic characteristics.

Variable	Control (n=30)	Stable graft (n=25)	Rejection (n=20)	p-Value
Age, years, mean ± SD	37.3 ± 5.0	38.4 ± 5.6	38.9 ± 8.9	0.733
Age, years, median [IQR]	37 [33–41]	39 [35–42]	38 [33–46]	–
Sex, M/F	15/15	20/5	12/8	0.070
Transplant type, living/cadaveric	N/A	N/A	12/8	–
Creatinine at rejection, mg/dL, median [range]	N/A	N/A	6.05 [2.65–12.60]	–
Rejection subtype (TCMR/AMR)	N/A	N/A	11/9	–
DSA status, positive/negative	N/A	N/A	12/8	–

Age: Kruskal–Wallis. Sex: chi-square. p>0.05 for all between-group comparisons. IQR, interquartile range; N/A, not applicable.

### mRNA expression

**Figure 1:**
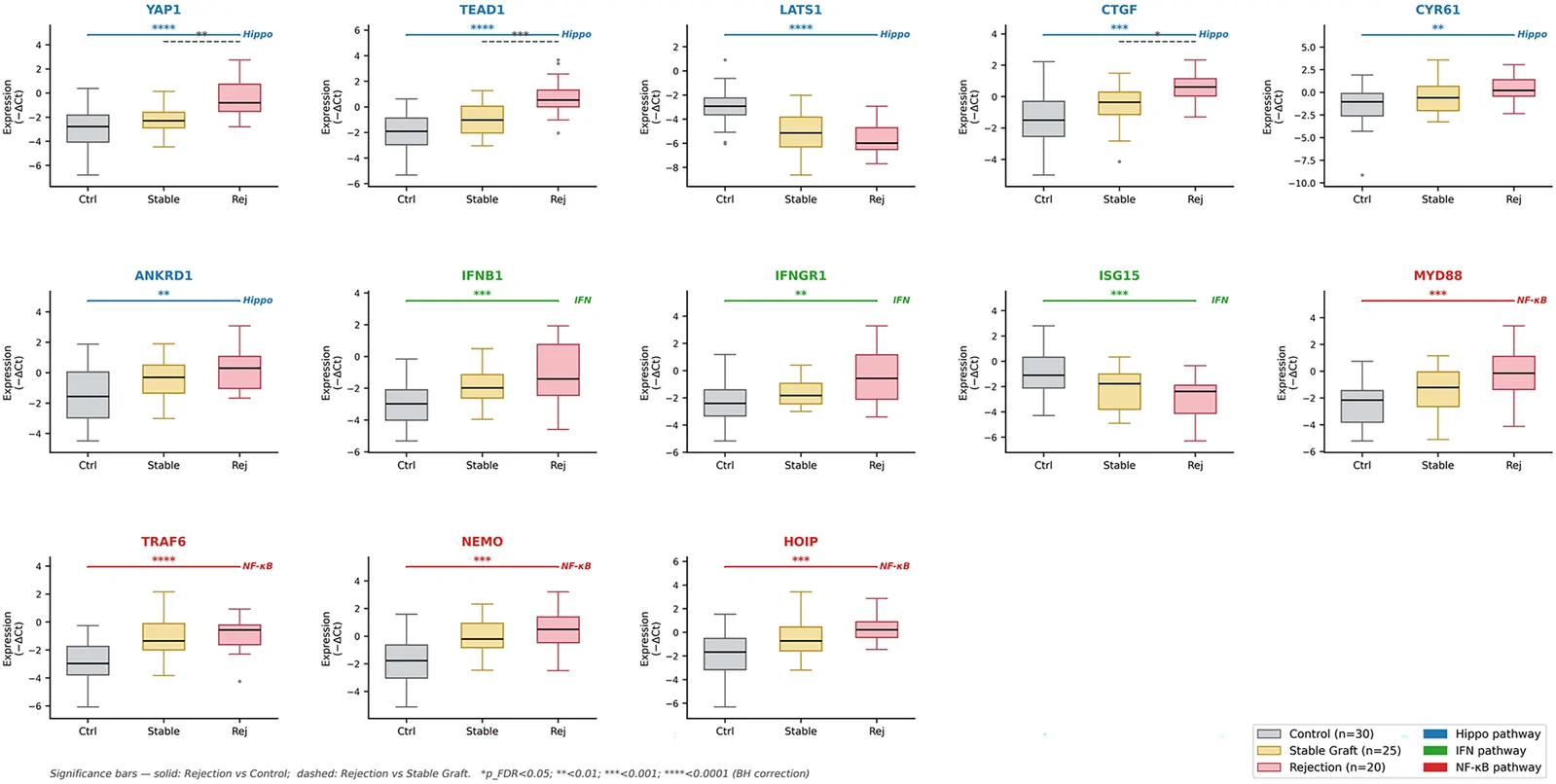
mRNA expression of 13 pathway genes in peripheral blood mononuclear cells. Box plots across three groups (control n=30, stable graft n=25, rejection n=20). Panels are arranged in a 5 + 5 + 3 grid, reading top-left to bottom-right: row 1 – *YAP1*, *TEAD1*, *LATS1*, *CTGF* and *CYR61* (Hippo pathway; panel titles in blue); row 2 – *ANKRD1* (Hippo; blue), *IFNB1*, *IFNGR1*, *ISG15* (interferon pathway; titles in green) and *MYD88* (NF-κB pathway; titles in red); row 3 – *TRAF6*, *NEMO* and *HOIP* (NF-κB; red). Box plots of *GAPDH*-normalised −ΔCt values; box, interquartile range; central line, median; whiskers, 1.5 × IQR; circles, outlying samples. Solid significance bars indicate rejection vs. control; dashed bars indicate rejection vs. stable graft. *pFDR<0.05; **pFDR<0.01; ***pFDR<0.001; ****pFDR<0.0001 (Benjamini–Hochberg correction).

Thirteen of 14 genes were significantly upregulated in rejection vs. controls (pFDR<0.01; Table 2; Figure 1). *TEAD1* (log_2_FC=+2.44; pFDR<0.0001) and *TRAF6* (+2.40; pFDR<0.0001) showed the largest increases; *YAP1* (+1.97), *MYD88* (+2.01), *NEMO* (+2.27), *CTGF* (+2.12), *HOIP* (+1.89), *HOIL-1* (+1.60) and *ANKRD1* (+1.87) were also elevated. *LATS1* was suppressed (log_2_FC=−3.06; pFDR<0.0001). The concurrent *LATS1* suppression and *YAP1*–*TEAD1* elevation constitutes the Hippo-OFF transcriptional pattern [4], 5].

**Table 2:** mRNA expression – three pairwise comparisons (log_2_ fold-change, pFDR).

Gene	Pathway	Stable vs. control	Rejection vs. control	Rejection vs. stable
CTGF	Hippo	+1.14^d^	+2.12^b^	+0.97^d^
CYR61	Hippo	+0.45 ns	+1.25^c^	+0.80 ns
IFNB1	IFN	+1.00^d^	+1.57^b^	+0.57 ns
IFNGR1	IFN	+0.58 ns	+1.84^c^	+1.26 ns
ISG15	IFN	−0.66^d^	−1.28^b^	−0.62 ns
MYD88	NF-κB	+0.94^d^	+2.01^b^	+1.07 ns
TRAF6	NF-κB	+1.61^c^	+2.40^a^	+0.79 ns
NEMO	NF-κB	+1.57^b^	+2.27^b^	+0.70 ns
HOIP	NF-κB	+0.94^d^	+1.89^b^	+0.94 ns
HOIL-1	NF-κB	+0.68^d^	+1.60^b^	+0.32 ns
ANKRD1	Hippo	+1.26^d^	+1.87^c^	+0.61 ns
TEAD1	Hippo	+0.88^d^	+2.44^a^	+1.56^a^
YAP1	Hippo	+0.49 ns	+1.97^a^	+1.49^b^
LATS1	Hippo	−2.22^b^	−3.06^a^	−0.84 ns

Mann–Whitney U; Benjamini–Hochberg false-discovery-rate correction. ^a^p<0.0001; ^b^p<0.001; ^c^p<0.01; ^d^p<0.05; ns, non-significant. Negative values for ISG15 and LATS1 indicate downregulation. Gene names follow HUGO nomenclature.

*HOIL-1*, the LUBAC regulatory subunit not previously reported alongside *HOIP* in this cohort, showed a near-identical pattern: significantly elevated in stable graft (log_2_FC=+0.68; pFDR<0.05) and rejection (+1.60; pFDR<0.001) vs. controls, but not different between rejection and stable graft (+0.32; ns; Table 2). The concordance between *HOIP* and *HOIL-1* supports coordinated transcriptional regulation of the LUBAC complex as a unit, addressing the concern that single-subunit data cannot speak to complex-level integrity.

*ISG15* alone was downregulated in both rejection vs. controls (log_2_FC=−1.28; pFDR<0.001) and stable graft vs. controls (−0.66; pFDR<0.05). *IFNB1* (+1.57) and *IFNGR1* (+1.84) were elevated in rejection. The divergence between elevated upstream IFN components and the suppressed downstream effector points to post-receptor modulation of IFN-stimulated gene transcription by calcineurin inhibitor therapy [23], 24].

In the stable graft group, 12 of 14 genes showed intermediate activation between controls and rejection (Table 2). *LATS1* suppression was already substantial in stable graft vs. controls (log_2_FC=−2.22; pFDR<0.001), indicating that loss of Hippo transcriptional restraint is a feature of the post-transplant state broadly, not a marker of active rejection.

In the rejection-versus-stable graft comparison, only *TEAD1* (log_2_FC=+1.56; pFDR<0.001; Cohen’s *d*=1.40; power=0.997) and *YAP1* (+1.49; pFDR<0.01; *d*=1.17; power=0.973) remained significant, with *CTGF* borderline (+0.97; pFDR<0.05; *d*=0.89; power=0.843).

### Serum biomarkers

**Figure 2:**
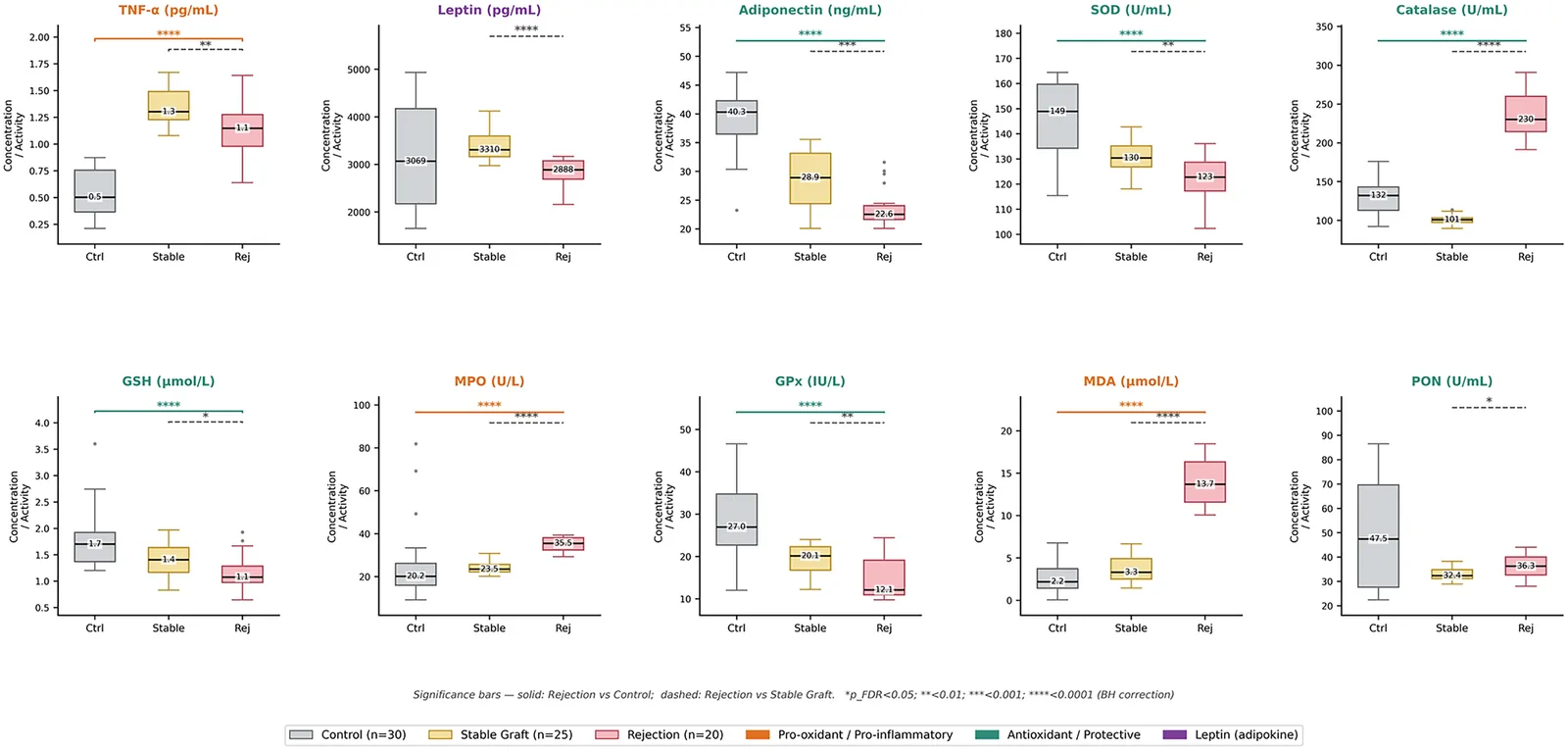
Serum biomarker comparison across the three groups. Box plots (control n=30, stable graft n=25, rejection n=20). Panels are arranged in a 5 + 5 grid, reading top-left to bottom-right: row 1 – TNF-α (pg/mL; pro-inflammatory, orange edge), leptin (pg/mL; adipokine, purple edge), adiponectin (ng/mL; protective adipokine, teal edge), SOD (U/mL; antioxidant, teal) and catalase (U/mL; antioxidant, teal); row 2 – GSH (µmol/L; antioxidant, teal), MPO (U/L; pro-oxidant, orange), GPx (IU/L; antioxidant, teal), MDA (µmol/L; oxidative-stress marker, orange) and PON (U/mL; antioxidant, teal). Box plots with median annotated inside each box; whiskers, 1.5 × IQR; circles, outlying samples. Solid significance bar indicates rejection vs. control; dashed bar indicates rejection vs. stable graft. *pFDR<0.05; **pFDR<0.01; ***pFDR<0.001; ****pFDR<0.0001 (Benjamini–Hochberg correction).

Ten of 11 serum parameters changed significantly in rejection vs. controls (Table 3; Figure 2). Catalase (230.2 vs. 132.3 U/mL), MDA (13.7 vs. 2.2 μmol/L) and MPO (35.6 vs. 20.2 U/L) were markedly elevated. SOD, GPx, GSH and adiponectin decreased, indicating impaired antioxidant capacity. Serum free ISG15 protein was essentially unchanged between stable graft and controls (144.4 vs. 150.4 ng/mL; pFDR, ns) but was markedly lower in rejection than in both controls (80.1 vs. 150.4 ng/mL; pFDR<0.001) and stable graft (80.1 vs. 144.4 ng/mL; pFDR=0.002) – a rejection-versus-stable discrimination that PBMC ISG15 mRNA did not achieve (Table 2), suggesting that circulating ISG15 protein carries diagnostic information beyond the transcriptional signal. TNF-α was lower in rejection than in stable graft (1.15 vs. 1.30 pg/mL; pFDR=0.006) – a pattern consistent with acute suppression by pulse corticosteroids and lymphocyte-depleting agents [12]. PON did not differ between rejection and controls (pFDR=0.174) but differed between rejection and stable graft (pFDR=0.017).

**Table 3:** Serum biomarkers – three-group comparison (median values).

Parameter	Control	Stable	Rejection	Sta–Ctrl pFDR	Rej–Ctrl pFDR	Rej–Sta pFDR
TNF-α	0.50	1.30	1.15	<0.0001^a^	<0.0001^a^	0.006^c^
Leptin	3,069.21	3,309.80	2,887.83	0.220 ns	0.714 ns	<0.0001^a^
Adiponectin	40.33	28.93	22.55	<0.0001^a^	<0.0001^a^	0.0006^b^
SOD	148.95	130.42	122.81	0.0001^b^	<0.0001^a^	0.008^c^
Catalase	132.26	100.98	230.21	<0.0001^a^	<0.0001^a^	<0.0001^a^
GSH	1.70	1.41	1.08	0.005^c^	<0.0001^a^	0.011^d^
MPO	20.19	23.51	35.55	0.042^d^	<0.0001^a^	<0.0001^a^
GPx	26.97	20.14	12.10	0.0002^b^	<0.0001^a^	0.001^c^
MDA	2.20	3.32	13.70	0.013^d^	<0.0001^a^	<0.0001^a^
PON	47.48	32.42	36.32	0.064 ns	0.174 ns	0.017^d^
ISG15, ng/mL	150.4 (60.8–200.7)	144.4 (55.8–194.7)	80.1 (51.7–159.7)	0.041 ns	<0.001^a^	0.002^c^

Units: TNF-α (pg/mL); leptin (pg/mL); adiponectin (ng/mL); SOD (U/mL); catalase (U/mL); GSH (µmol/L); MPO (U/L); GPx (IU/L); MDA (µmol/L); PON (U/mL). ^a^p<0.0001; ^b^p<0.001; ^c^p<0.01; ^d^p<0.05; ns, non-significant.

### Diagnostic performance – rejection vs. stable graft

**Figure 3:**
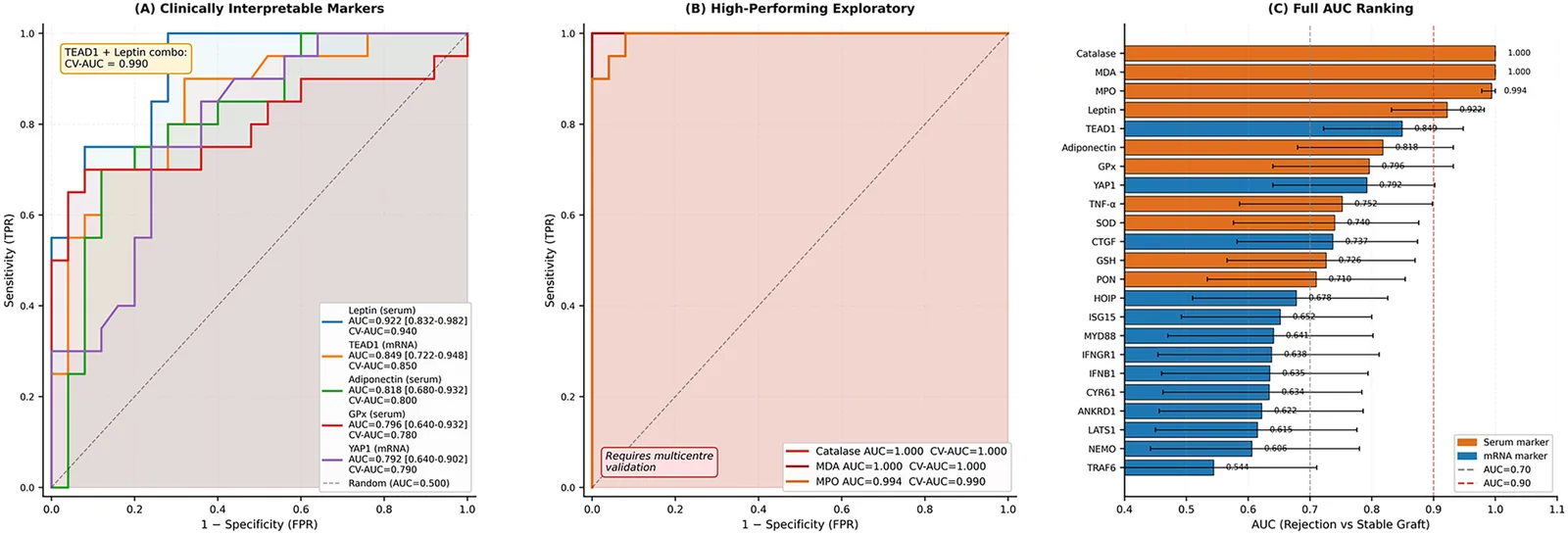
Receiver operating characteristic analysis, rejection vs. stable graft (n=20 vs. n=25). (A) The five highest-ranking clinically interpretable single markers: leptin, TEAD1 mRNA, adiponectin, GPx and YAP1 mRNA. Of these, only leptin and TEAD1 have a 95 % confidence interval lying entirely above AUC=0.70. (B) High-performing serum markers (AUC≈1.000; designated exploratory single-centre): catalase, MDA and MPO. (C) Full AUC ranking for all 23 markers with 2,000-iteration bootstrap 95 % CIs. AUC=0.70 and 0.90 reference lines are shown.

**Figure 4:**
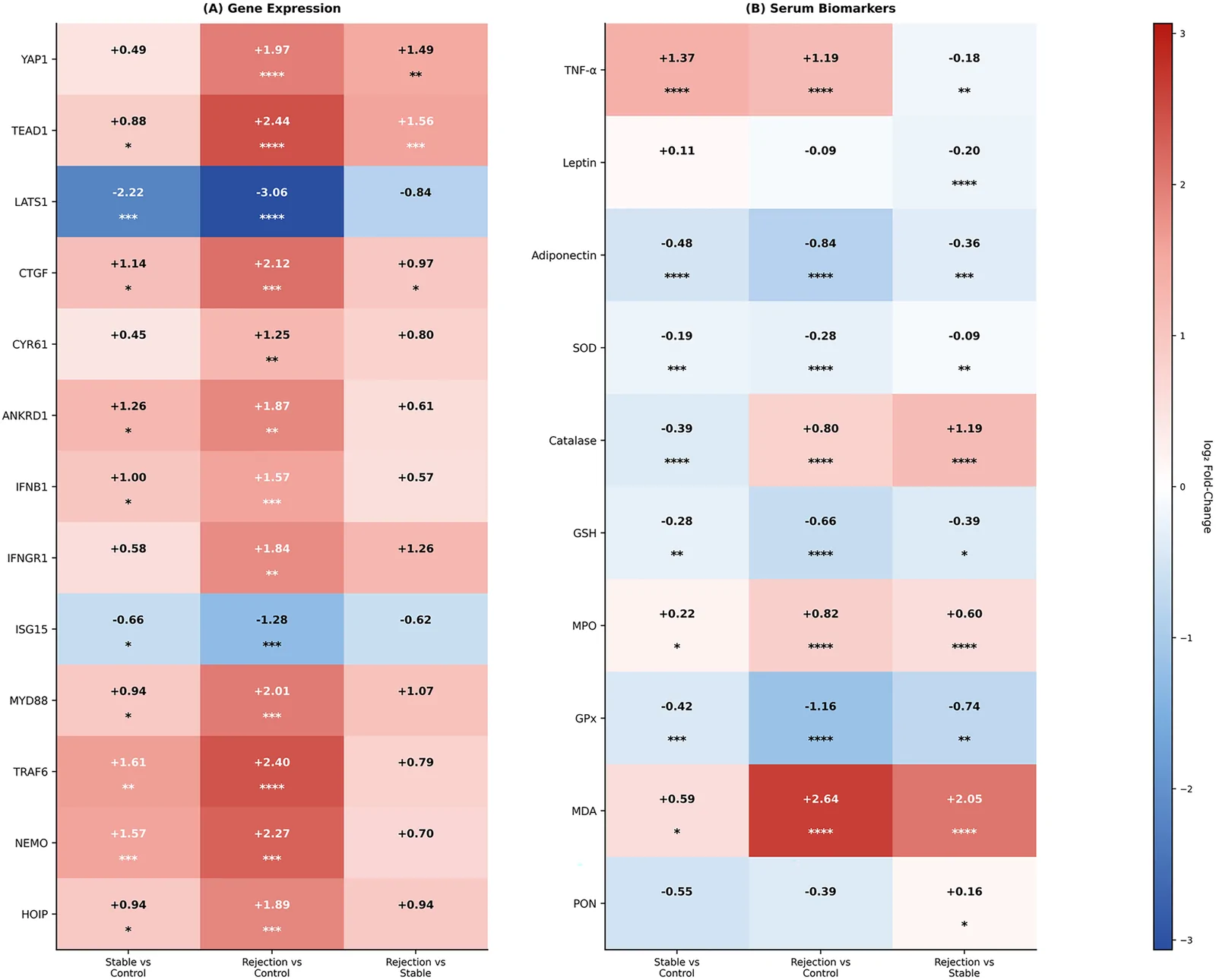
Log_2_ fold-change heatmap. (A) Gene expression: 13 panel genes across three pairwise comparisons (stable vs. control; rejection vs. control; rejection vs. stable). (B) Serum biomarkers: 10 parameters across the same three comparisons. Red, upregulation; blue, downregulation. SG15 is the sole downregulated IFN pathway gene in both transplant groups.

MDA (AUC=1.000; CV-AUC=1.000) and catalase (AUC=1.000) are designated exploratory (see Discussion). MPO showed AUC=0.994 [0.976–1.000; CV-AUC=0.994]. Among markers whose 95 % CIs fully exclude AUC=0.70, leptin (AUC=0.922 [0.833–0.980]; lower in rejection) and *TEAD1* mRNA (AUC=0.849 [0.724–0.948]; higher in rejection) are the strongest single candidates. *YAP1* mRNA gave AUC=0.792 [0.644–0.913]. A *TEAD1* + leptin combination yielded a cross-validated AUC of 0.990 (Table 4; Figure 3). Log₂ fold-changes for all genes and serum parameters across the three pairwise comparisons are summarised in Figure 4.

**Table 4:** ROC analysis – rejection Vs. stable graft.

Rank	Marker	Type	AUC	95 % CI	CV-AUC	Direction
1	MDA	Serum	1.000	[1.000–1.000]	1.000	Rej>Sta
2	Catalase	Serum	1.000	[1.000–1.000]	1.000	Rej>Sta
3	MPO	Serum	0.994	[0.976–1.000]	0.994	Rej>Sta
4	Leptin	Serum	0.922	[0.833–0.980]	0.916	Rej<Sta
5	TEAD1	mRNA	0.849	[0.724–0.948]	0.824	Rej>Sta
6	Adiponectin	Serum	0.818	[0.680–0.929]	0.800	Rej<Sta
7	GPx	Serum	0.796	[0.630–0.939]	0.790	Rej<Sta
8	YAP1	mRNA	0.792	[0.644–0.913]	0.788	Rej>Sta
9	TNF-α	Serum	0.752	[0.583–0.894]	0.718	Rej<Sta
10	SOD	Serum	0.740	[0.571–0.879]	0.738	Rej<Sta
11	CTGF	mRNA	0.737	[0.582–0.872]	0.719	Rej>Sta
12	GSH	Serum	0.726	[0.563–0.875]	0.672	Rej<Sta

AUC 95 % CI: 2,000-iteration stratified bootstrap. CV-AUC: 5-fold stratified cross-validation. Catalase and MDA AUC=1.000 are designated exploratory (see Discussion). Direction: Rej>Sta, higher in rejection; Rej<Sta, lower in rejection.

### Banff histopathological correlations

**Figure 5:**
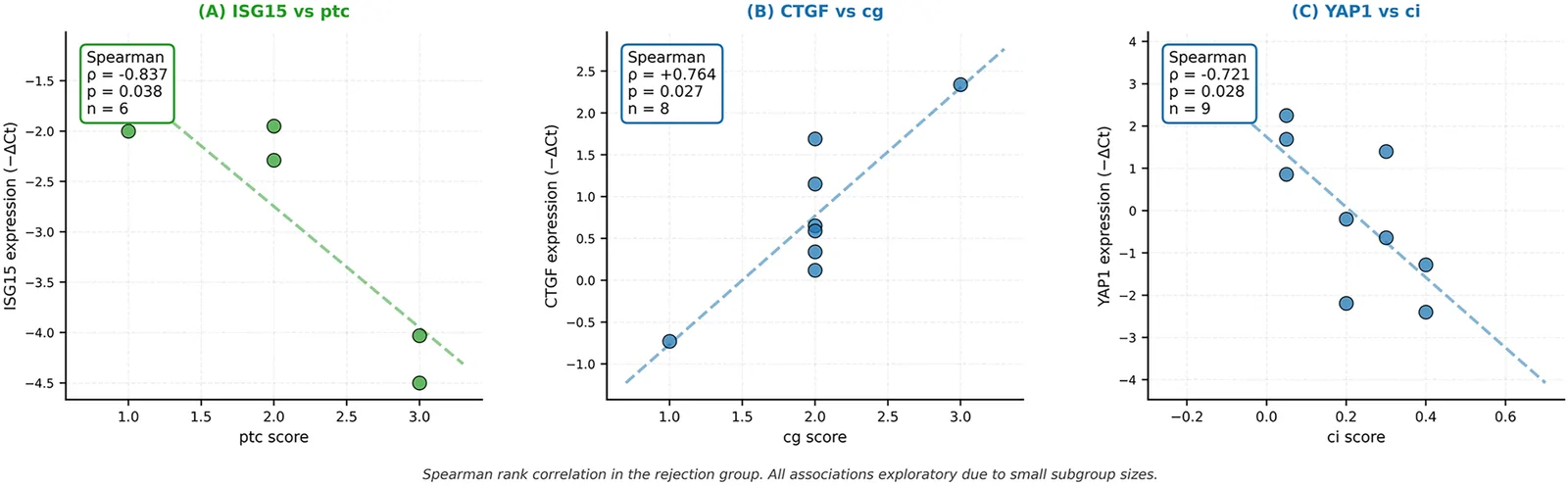
Banff histopathological score vs. gene expression in the rejection group; Spearman rank correlation. (A) *ISG15* vs. peritubular capillaritis (ptc) score: ρ=−0.837; p=0.038; n=6. (B) *CTGF* vs. GBM double-contour (cg) score: ρ=+0.764; p=0.027; n=8. (C) *YAP1* vs. interstitial fibrosis (ci) score: ρ=−0.721; p=0.028; n=9. All associations are exploratory due to small subgroup sizes.

Four significant Spearman associations (|ρ|>0.5; p<0.05) were identified in the rejection group (Table 5; Figure 5). *ISG15* was negatively associated with peritubular capillaritis score (ptc; ρ=−0.837; p=0.038; n=6). GBM double contour (cg) correlated positively with *CTGF* (ρ=+0.764; p=0.027; n=8). *YAP1* and *LATS1* were both negatively associated with interstitial fibrosis (ci; ρ=−0.721 and −0.687; n=9). All associations are exploratory given the small subgroup sizes.

**Table 5:** Banff score – gene expression correlations (rejection group; exploratory).

Banff parameter	Gene	Spearman ρ	p-Value	n
Peritubular capillaritis (ptc)	ISG15	−0.837	0.038	6
GBM double contour (cg)	CTGF	+0.764	0.027	8
Interstitial fibrosis (ci)	YAP1	−0.721	0.028	9
Interstitial fibrosis (ci)	LATS1	−0.687	0.041	9

Only |ρ|>0.5 and p<0.05 are listed. All correlations are exploratory due to small subgroup sample sizes (n=6–9). External validation in larger cohorts is required.

### Directional cross-validation in public datasets

**Figure 6:**
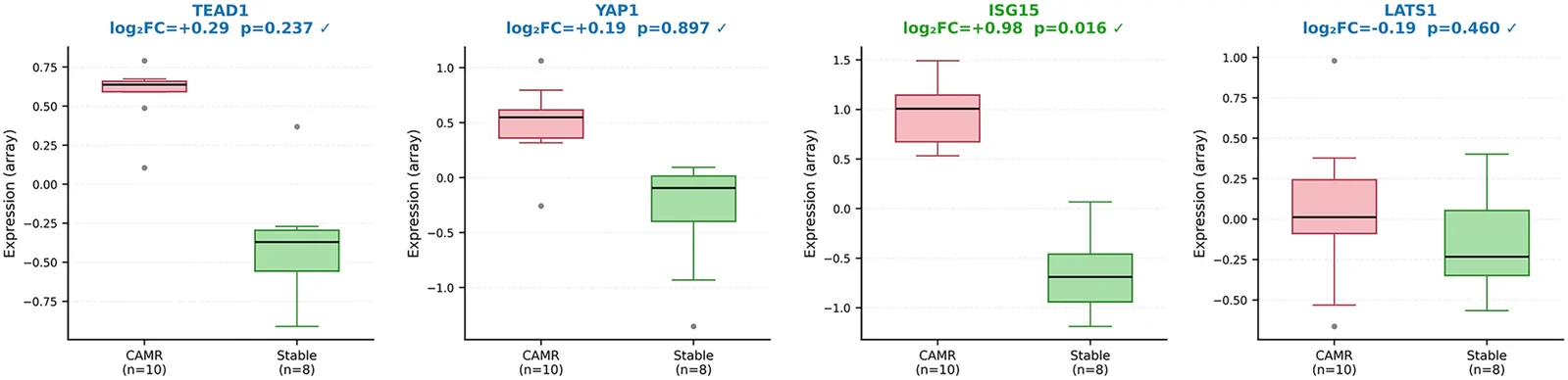
External directional validation in GSE51675. Agilent GPL6480 microarray; peripheral blood mononuclear cell expression data from chronic antibody-mediated rejection (CAMR; n=10) vs. stable graft recipients (n=8). Panels are arranged in a single row, reading left to right: *TEAD1*, *YAP1*, *ISG15* and *LATS1*. The log_2_ fold-change and Mann–Whitney *U* p value are shown above each panel; the ✓ symbol indicates direction consistent with our primary qRT-PCR cohort. *TEAD1*, *YAP1* and *LATS1* show directionally consistent changes with the primary cohort; *ISG15* differs in direction here (CAMR>stable) because both groups are immunosuppressed (see Discussion). All comparisons are non-significant after FDR correction, as expected for n=8–10.

In GSE51675 (CAMR vs. stable PBMC; n=10 vs. n=8; Figure 6), *TEAD1* (log_2_FC=+0.29), *YAP1* (+0.19) and *LATS1* (−0.19) all moved in the same direction as our qRT-PCR cohort. *MYD88* (+0.34) and *TRAF6* (+0.09) were numerically higher in CAMR but non-significant after FDR correction – the same transplantation-associated NF-κB pattern seen in our primary data. *ISG15* was higher in CAMR than stable (+0.98, ns); both groups in GSE51675 are immunosuppressed, so this CAMR-versus-stable *ISG15* direction reflects local IFN activity differences within the transplant population rather than the immunosuppression effect we identify against healthy controls. None of the GSE51675 comparisons reached significance after correction, as expected at n=8–10.

**Figure 7:**
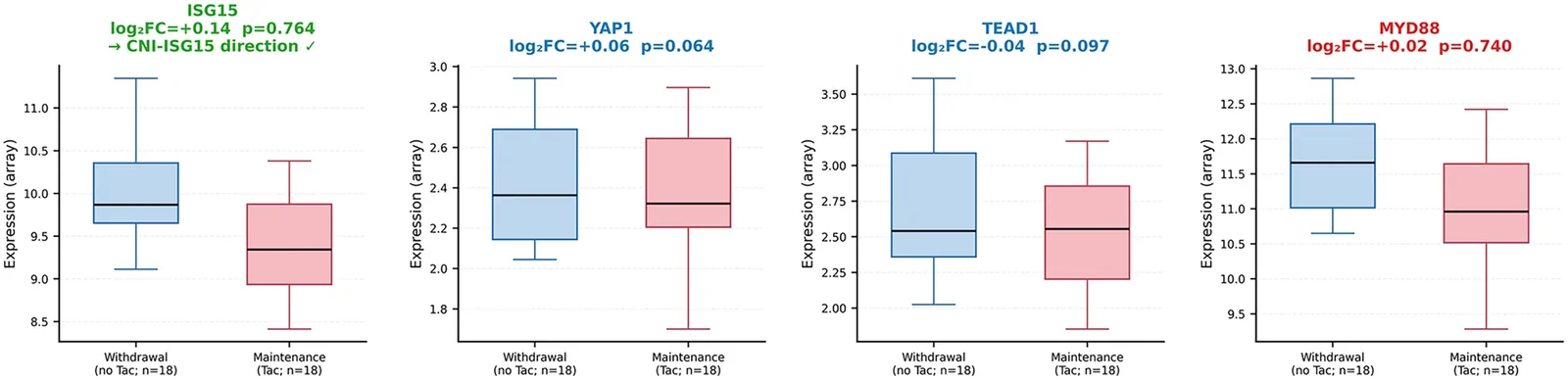
External directional validation in GSE145408. Affymetrix GPL13158 microarray; CTOT-09 trial, peripheral blood mononuclear cell expression data from kidney transplant recipients randomised to tacrolimus withdrawal (no Tac; n=18) vs. tacrolimus maintenance (Tac; n=18); paired design. Panels are arranged in a single row, reading left to right: *ISG15*, *YAP1*, *TEAD1* and *MYD88*. The log_2_ fold-change (withdrawal vs. maintenance) and Mann–Whitney *U* p value are shown above each panel. *ISG15* shows a numerical increase after tacrolimus removal (log_2_FC=+0.14; p=0.764), directionally consistent with calcineurin inhibitor-mediated *ISG15* suppression. Effect is not significant.

In GSE145408 (tacrolimus withdrawal vs. maintenance; n=18 each; paired design; Figure 7), *ISG15* was numerically higher after tacrolimus removal (log_2_FC=+0.14; p=0.764). The direction is consistent with CNI-mediated suppression – remove the drug, *ISG15* increases – but the effect size is small. Stable patients on partial residual immunosuppression over a three-month follow-up would not be expected to show full derepression seen acutely. *YAP1* showed a borderline increase after withdrawal (+0.06; p=0.064). Full results are in Supplementary Table S4 and summarised in Table 6.

**Table 6:** Directional cross-validation summary.

Gene	Our cohort (Rej vs. Sta)	GSE51675 (CAMR vs. Sta)	Direction consistent	GSE145408 (W vs. M)	Interpretation
TEAD1	+1.56^a^	+0.29 ns	Yes	−0.04 ns	Hippo rejection marker
YAP1	+1.49^b^	+0.19 ns	Yes	+0.06 ns	Hippo rejection marker
LATS1	−0.81 ns	−0.19 ns	Yes	−0.07 ns	Hippo suppressor
ISG15	−0.62 ns	+0.98 ns	Mixed	+0.14 ns	CNI–ISG15 direction
MYD88	+0.01 ns	+0.34 ns	Yes	–	Transplant-associated
TRAF6	+0.79 ns	+0.09 ns	Yes	–	Transplant-associated

Our cohort and GEO datasets: Mann–Whitney U with Benjamini–Hochberg correction. W vs. M=withdrawal vs. maintenance (GSE145408). ISG15 direction differs between comparisons: in our cohort, both transplant groups are lower than healthy controls (immunosuppression effect); in GSE51675, CAMR>stable (both immunosuppressed; CAMR has more active IFN signalling); in GSE145408, withdrawal>maintenance, supporting the CNI–ISG15 direction. These three comparisons measure different axes of the same pharmacological-immunological relationship. ^a^pFDR<0.001; ^b^pFDR<0.01.

## Discussion

Three findings from this cohort stand out. *TEAD1* and *YAP1* were the only genes that distinguished rejection from stable graft with statistical power adequate for a reliable inference – and both directions replicated in an independent microarray dataset. *ISG15* was suppressed across both transplant groups rather than elevated – a pharmacological rather than immunological signal, directionally supported by the tacrolimus withdrawal data. The stable graft group occupied a molecular middle ground between controls and rejection, which argues against the assumption that successful transplantation means complete immune quiescence.

### Hippo pathway in PBMC: immune cell activation, not intragraft biology

*LATS1* suppression (log_2_FC=−3.06) together with *YAP1* (+1.97) and *TEAD1* (+2.44) elevation defines the Hippo-OFF transcriptional pattern documented in renal fibrosis [4], [5], [6]. The data come from PBMCs – immune cells, not tubular epithelium or podocytes – so the signal reflects immune cell-intrinsic *YAP1*–*TEAD1* programmes: Th17 differentiation [8], macrophage inflammatory reprogramming [9], and effector T cell survival. Whether peripheral PBMC Hippo activation tracks intragraft Hippo status requires parallel tissue–PBMC sampling, which was not performed here.

*LATS1* mRNA suppression does not equate to kinase inactivation. LATS1 activity is set post-translationally by MST1/2-dependent phosphorylation at Thr1079; mRNA data reflect transcriptional dysregulation, not confirmed nuclear YAP1 translocation [4]. Despite this, *TEAD1* and *YAP1* have the largest effect sizes in the rejection-versus-stable graft comparison (*d*=1.40 and 1.17; power=0.997 and 0.973), and their direction replicated in GSE51675 (*TEAD1* +0.29, *YAP1* +0.19 in CAMR vs. stable), which used a different microarray platform and patient population. *LATS1* suppression was already near-maximal in stable graft recipients (log_2_FC=−2.22 vs. controls), suggesting that the loss of Hippo transcriptional restraint is a general post-transplant feature rather than a rejection-specific event. What distinguishes rejection is the magnitude of downstream *YAP1*–*TEAD1* activation, not whether the pathway is on or off.

### NF-κB genes: elevated in transplantation, not specific to rejection

**Figure 8:**
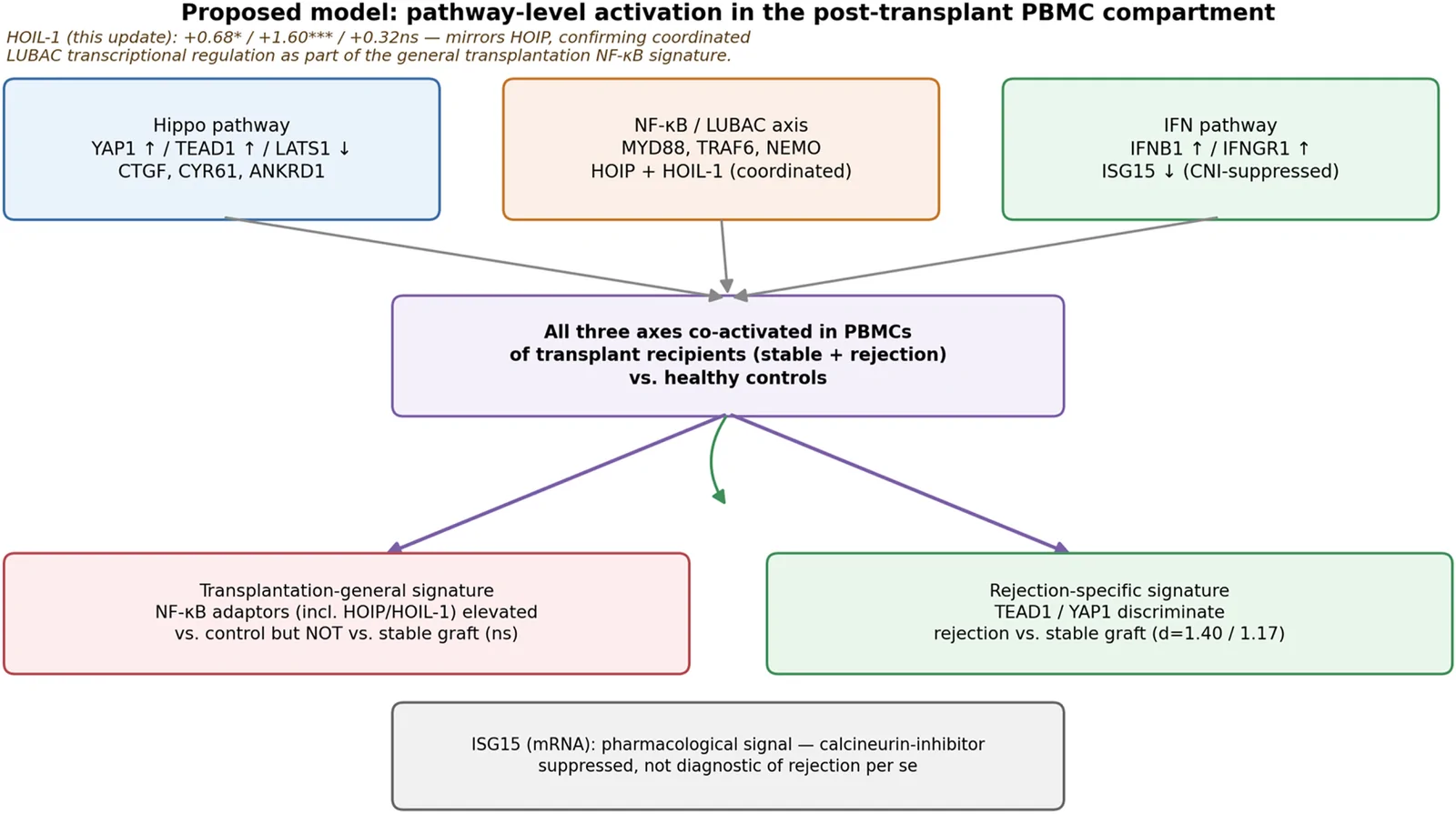
Proposed model of pathway-level activation in the post-transplant PBMC compartment. Schematic summary hypothesis integrating the present findings. Hippo, NF-κB/LUBAC and IFN pathway transcripts are jointly elevated in PBMCs of transplant recipients (stable graft and rejection) relative to healthy controls. Within this shared transplantation-associated signature, two sub-patterns diverge: (i) NF-κB adaptor genes, including the LUBAC subunits *HOIP* and *HOIL-1*, are elevated vs. controls but do not discriminate rejection from stable graft, marking the general transplantation state rather than active rejection; and (ii) Hippo effectors *TEAD1* and *YAP1* retain adequate discriminatory power between rejection and stable graft. *ISG15* mRNA behaves as a separate, calcineurin-inhibitor-sensitive pharmacological signal rather than a rejection marker. The near-identical fold-change and significance pattern between *HOIP* (rejection vs. control log_2_FC=+1.89, p<0.001; rejection vs. stable=+0.94, ns) and *HOIL-1* (+1.60, p<0.001; +0.32, ns) supports coordinated transcriptional regulation of the LUBAC complex as a single functional unit rather than independent single-gene variation. This model is hypothesis-generating; protein-level and functional validation are proposed as follow-up work (see Limitations).

*MYD88*, *TRAF6*, *NEMO*, *HOIP* and *HOIL-1* were all elevated in rejection vs. controls but none reached significance in rejection vs. stable graft. The same pattern held in GSE51675 (*MYD88* +0.34, ns; *TRAF6* +0.09, ns). Sharbafi et al. [25] reported the same phenomenon for *MYD88* across acute, chronic and stable graft states; our data extend that observation to the full *MYD88*–*TRAF6*–*NEMO*–*HOIP*/*HOIL-1* adaptor architecture. Because LUBAC-mediated linear ubiquitination of NEMO requires both the catalytic (HOIP) and regulatory (HOIL-1) subunits for complex stability and full activity [10], measuring HOIP alone leaves LUBAC integrity only partially characterised; the near-identical fold-change and significance pattern we observe for *HOIP* and *HOIL-1* (Table 2; Figure 8) indicates the two subunits are co-regulated at the transcript level, consistent with coordinated LUBAC complex upregulation rather than an isolated single-gene effect. This strengthens the case that LUBAC engagement is part of the general NF-κB transplantation signature, while reinforcing – rather than resolving – the point that mRNA co-regulation is not proof of complex assembly or catalytic activity, which requires protein-level confirmation (Limitations). NF-κB PBMC gene panels measure the transplantation state, not the rejection state, so for the practical question of whether rejection is occurring in a given patient these genes would not add discriminatory value. Tacrolimus further confounds the interpretation by suppressing NF-κB activation in T cells through calcineurin inhibition [24], a drug effect that cannot be quantified without regimen data.

### ISG15: a candidate pharmacological signal, not yet a confirmed rejection marker

*ISG15* was the only gene downregulated in both transplant groups relative to healthy controls, while *IFNB1* and *IFNGR1* were elevated in rejection – the same IFN pathway, diverging at the receptor–effector junction. This is not a paradox once the drug regimen is considered. Calcineurin inhibitors block NFAT-dependent IFN-γ transcription in T cells [12]; in solid organ transplant recipients, tacrolimus suppresses IFN-γ-producing PBMC T cells in a concentration-dependent manner at clinically achieved intra-PBMC levels [13]. *ISG15* transcription is driven by JAK–STAT1/2 downstream of IFN-γ; reducing IFN-γ under CNI would be expected to lower *ISG15* expression even if the IFN receptor remains at the cell surface, although this pathway-level inference was not directly tested in the present cohort. *ISG15* also negatively regulates IFN–STAT1 signalling in regulatory T cells [26], so CNI-mediated changes may extend beyond simple cytokine depletion.

The tacrolimus withdrawal data from GSE145408 are consistent with this picture: *ISG15* trended upward after tacrolimus removal (+0.14; p=0.764). The effect was small – stable patients on partial immunosuppression over three months would not be expected to show dramatic changes – but the direction matches. We draw no quantitative conclusions from this dataset; the GSE145408 result is a directional check, not a replication. The absence of immunosuppressive regimen data in our own cohort remains the primary limitation that prevents direct testing of the dose–response relationship. Future studies should collect CNI trough levels prospectively alongside PBMC sampling. The negative association between *ISG15* and peritubular capillaritis score (ρ=−0.837; n=6; exploratory) fits this framework: patients with more active microvascular rejection are likely on more aggressive immunosuppression, further suppressing *ISG15*.

Serum free *ISG15* protein showed a different, and diagnostically more informative, pattern than the PBMC transcript. Serum ISG15 was essentially unchanged between stable graft and controls (144.4 vs. 150.4 ng/mL; pFDR, ns) but was markedly lower in rejection than in both controls (80.1 vs. 150.4 ng/mL; pFDR<0.001) and stable graft (80.1 vs. 144.4 ng/mL; pFDR=0.002) – a rejection-versus-stable discrimination that PBMC *ISG15* mRNA did not achieve (Table 2). This transcript–protein discordance could reflect several non-mutually-exclusive mechanisms: additional post-transcriptional or post-translational regulation of ISG15 secretion and turnover specific to active rejection; a contribution of graft-derived or non-PBMC systemic sources to circulating ISG15 that PBMC mRNA does not capture; or greater sensitivity of the circulating protein pool to the more intensive immunosuppressive regimens typically used to treat acute rejection episodes, superimposed on the chronic calcineurin inhibitor effect already visible at the transcript level. We cannot adjudicate between these explanations without immunosuppressive regimen and dosing data, which were not systematically available in this cohort. Regardless of mechanism, the practical implication is that a purely transcriptional ISG15 assay would have missed the strongest single serum discriminator of rejection vs. stable graft identified in this study, and that circulating ISG15 protein – rather than its PBMC transcript – appears to be the more diagnostically relevant readout and merits inclusion alongside TEAD1 mRNA and leptin in future prospective validation.

### Serum oxidative stress and the clinical candidates

Catalase, MDA and MPO achieved AUC≥0.994 for rejection vs. stable graft. The biology is coherent: MDA elevation indicates lipid peroxidation from alloimmune activation; catalase induction is a compensatory antioxidant response; MPO elevation reflects neutrophil-mediated damage. The concurrent falls in SOD, GPx and GSH show that compensation is inadequate. Adiponectin, an adipokine with anti-inflammatory and anti-fibrotic actions [27], and PON-1, an HDL-associated antioxidant enzyme [28], were also lower in rejection than in controls, although the PON-1 difference did not reach significance. These three markers are designated exploratory because single-centre batch conditions make it difficult to exclude analytical sources of the separation, and because immunosuppressive drug-dose differences between the groups – pulse corticosteroids in rejecting patients – could independently drive the oxidative stress profile. Multicentre validation using standardised assay conditions is needed.

Leptin (AUC=0.922; lower in rejection) most likely reflects corticosteroid pulse suppression of adipose leptin secretion [12] rather than rejection biology per se. *TEAD1* mRNA (AUC=0.849; *d*=1.40; power=0.997) remains the most defensible single candidate: the confidence interval is tight (0.724–0.948), the effect size is large, the power is adequate, and the direction replicated in an independent dataset.

### AUC=1.000: the numbers and what they mean

Catalase and MDA achieved AUC=1.000 with CV-AUC=1.000 in n=20 vs. n=25. Three factors prevent reading this as clinical performance: homogeneous single-centre assay batch conditions; no independent validation cohort; and potential immunosuppressive drug-dose differences between rejection and stable graft groups that are confounded with the biomarker signal. The markers are reported for completeness and to generate multicentre hypotheses, not as evidence of clinical utility. The actionable single-marker candidates – those with 95 % CIs excluding AUC=0.70 – are leptin (0.922; CI 0.833–0.980), *TEAD1* mRNA (0.849; CI 0.724–0.948), adiponectin (0.818; CI 0.680–0.929) and *YAP1* mRNA (0.792; CI 0.644–0.913).

### Limitations

The most consequential gap is the absence of individual-level immunosuppressive dosing data. All patients were maintained on a standard calcineurin inhibitor (predominantly tacrolimus)–mycophenolate–corticosteroid regimen, and time-since-transplant was comparable between the stable graft and rejection groups (both >6 months post-transplantation), so drug class and transplant duration do not confound the rejection-versus-stable comparison at the group level. However, individual trough levels, exact dosing, and steroid intensification were not systematically recorded, so within-group pharmacological variability and its contribution to *ISG15*, TNF-α, leptin and the oxidative stress profile cannot be quantitatively separated from rejection biology; future prospective studies must collect these variables alongside every blood draw. The PBMC compartment profiled here is not renal parenchyma, and compartmental cross-validation requires parallel biopsy–PBMC sampling. Single-centre design with n=20 vs. n=25 means genes with *d*<0.8 are underpowered and null results are inconclusive. Male predominance in stable graft (20 M/5 F; p=0.070) may affect leptin and adiponectin. Single reference gene (*GAPDH*) was used; multi-gene normalisation is planned. All Banff correlations are exploratory (n=6–9). The GEO cross-validation datasets are small (n=8–18) and provide directional checks only, not statistical replication; moreover, neither is a direct model of acute cellular rejection – GSE51675 reflects chronic, antibody-mediated pathology, and GSE145408 contains no rejection episodes at all – so both provide only indirect, hypothesis-supportive evidence, and prospective validation in an independent acute rejection cohort remains necessary. The *ISG15* effect of tacrolimus withdrawal in GSE145408 was small and non-significant, indicating direction but not magnitude. LUBAC complex integrity was assessed transcriptionally by adding *HOIL-1* mRNA alongside *HOIP*, but this remains an mRNA-level readout; protein-level confirmation of LUBAC assembly and catalytic activity (e.g. co-immunoprecipitation, linear ubiquitin chain detection) was not performed. Direct mechanistic validation of the remaining proposed pathway interpretations – including ISG15ylation profiling by Western blot and IP-mass spectrometry, ex vivo IFN ± calcineurin inhibitor stimulation, *in vitro* comparison of calcineurin inhibitors against other immunosuppressants, and functional Hippo pathway manipulation (YAP1/TEAD1 silencing or overexpression) – was beyond the funding and infrastructure scope of the present pilot study; these experiments are planned for a dedicated, adequately funded follow-up project and the corresponding pathway-level interpretations in this manuscript should accordingly be read as hypothesis-generating rather than mechanistically confirmed.

## Conclusions

Thirteen of 14 Hippo, IFN and NF-κB pathway genes were upregulated in rejection vs. healthy controls. *ISG15* was the exception – suppressed in both transplant groups, a pattern consistent with a pharmacological effect of calcineurin inhibitor therapy, whose direction (but not magnitude) was supported by tacrolimus withdrawal data from GSE145408; direct mechanistic confirmation was beyond the scope of this pilot study. In contrast, serum free ISG15 protein discriminated rejection from stable graft (pFDR=0.002) despite being unchanged at the mRNA level between these two groups, indicating that the protein measurement carries diagnostic information the transcript does not and should be considered a candidate marker in its own right. NF-κB adaptor genes tracked the transplantation state, not the rejection state. Only *TEAD1* mRNA (AUC=0.849; *d*=1.40; power=0.997) and *YAP1* (AUC=0.792; *d*=1.17; power=0.973) discriminated rejection from stable graft with adequate power – a directional result that held in GSE51675 across an independent patient population and microarray platform. Serum leptin (AUC=0.922) is the strongest non-molecular candidate, though it likely reflects immunosuppressive drug effects. Prospective multicentre validation with systematic collection of drug levels is the necessary next step.

## Supplementary Material

Supplementary Material
