# Supplementary material for: Coordinated Hippo, IFN and NF-κB pathway activation in kidney rejection
Source: Open Med (Wars). 2026 Sep 8;21(1):20261516. doi: 10.1515/med-2026-1516 (PMC13552812; doi:10.1515/med-2026-1516)
Supplement: Supplementary file 1 [file j_med-2026-1516_suppl_001.docx]

**Coordinated Hippo, IFN and NF-κB pathway activation in renal allograft rejection: mRNA, serum biomarkers and external validation**

***Supplementary materials***

**Authors:** Eda Balkan, Murat Kızılkaya, Mahmut Aktürk, Elif Demirci, Necip Altundaş.

**Corresponding author:** Prof. Dr. Eda Balkan — eda.diyarbakir@hotmail.com

*This document contains Supplementary Tables S1–S4 referenced in the main manuscript. All tables follow the three-line format and CCLM nomenclature standards.*

**Supplementary tables**

**Supplementary Table S1: GAPDH reference gene stability across the three groups.**

| **Parameter** | **Control (n = 30)** | **Stable Graft (n = 25)** | **Rejection (n = 20)** |
| --- | --- | --- | --- |
| Mean Ct (± SD) | 22.28 ± 1.06 | 22.27 ± 0.83 | 22.14 ± 0.94 |
| Coefficient of variation (%) | 4.78 | 3.74 | 4.26 |
| Median Ct [IQR] | 22.3 [21.6–22.9] | 22.2 [21.7–22.8] | 22.1 [21.5–22.7] |
| Min – Max Ct | 20.4 – 24.6 | 20.8 – 23.9 | 20.6 – 24.1 |
| Kruskal–Wallis p (across groups) | — | — | 0.776 |

*Stability assessed by quantitative reverse transcription PCR. All groups showed coefficient of variation below 5 %, supporting GAPDH suitability as single reference gene. Kruskal–Wallis test was used for three-group comparison of mean Ct values; non-parametric due to small group sizes. Ct, threshold cycle; IQR, interquartile range; SD, standard deviation.*

**Supplementary Table S2: Human leucocyte antigen (HLA) mismatch profiles in the rejection group (n = 20).**

| **HLA mismatch profile** | **n (rejection group)** |
| --- | --- |
| 1A, 1B, 1DR | 7 |
| 1B, 1DR | 3 |
| 2DR | 3 |
| 1DR | 2 |
| 1A | 2 |
| 1A, 1DR | 1 |
| 2A, 2B, 2DR | 1 |
| Total | 20 |

*HLA mismatches recorded at the time of transplantation. The highest single-category frequency was the combined 1A + 1B + 1DR mismatch (n = 7; 35 %), followed by the 1B + 1DR and 2DR profiles (n = 3 each; 15 %). HLA, human leucocyte antigen.*

**Supplementary Table S3: Post-hoc statistical power analysis — rejection versus stable graft (n = 20 vs n = 25; two-tailed α = 0.05).**

| **Gene** | **Cohen's d** | **Observed power (1−β)** | **Interpretation** |
| --- | --- | --- | --- |
| *TEAD1* | 1.40 | 0.997 | Adequately powered |
| *YAP1* | 1.17 | 0.973 | Adequately powered |
| *CTGF* | 0.89 | 0.843 | Adequately powered |
| *IFNGR1* | 0.69 | 0.631 | Moderate power |
| *IFNB1* | 0.62 | 0.536 | Underpowered |
| *MYD88* | 0.60 | 0.511 | Underpowered |
| *HOIP* | 0.59 | 0.500 | Underpowered |
| *ISG15* | 0.55 | 0.455 | Underpowered |
| *ANKRD1* | 0.47 | 0.345 | Underpowered |
| *CYR61* | 0.46 | 0.330 | Underpowered |
| *LATS1* | 0.38 | 0.248 | Underpowered |
| *NEMO* | 0.35 | 0.218 | Underpowered |
| *TRAF6* | 0.17 | 0.087 | Underpowered |

*Cohen's d calculated from group means and pooled standard deviations. Power calculated for an independent two-sample t-test approximation given the observed effect sizes. Genes with d ≥ 0.8 are considered adequately powered; d 0.5–0.79 moderate power; d < 0.5 underpowered. Null findings for underpowered genes in the rejection-versus-stable graft comparison should be treated as inconclusive rather than negative.*

**Supplementary Table S4: Full directional cross-validation results across the primary cohort and two external Gene Expression Omnibus (GEO) datasets.**

| **Gene** | **Our cohort (Rej vs Sta)** | **GSE51675 (CAMR vs Sta)** | **GSE145408 (W vs M)** | **Direction** |
| --- | --- | --- | --- | --- |
| *TEAD1* | +1.56 *** | +0.29 ns | −0.04 ns | ↑ |
| *YAP1* | +1.49 ** | +0.19 ns | +0.06 ns | ↑ |
| *LATS1* | −0.81 ns | −0.19 ns | −0.07 ns | ↓ |
| *ISG15* | −0.62 ns | +0.98 ns | +0.14 ns | Mixed |
| *MYD88* | +0.01 ns | +0.34 ns | +0.02 ns | ↑ |
| *TRAF6* | +0.79 ns | +0.09 ns | +0.10 ns | ↑ |
| *ANKRD1* | +0.66 ns | +0.55 ns | N/A | ↑ |
| *CTGF* | +0.97 * | −0.24 ns | N/A | Mixed |
| *CYR61* | +0.25 ns | +0.19 ns | N/A | ↑ |
| *IFNB1* | +0.57 ns | −0.34 ns | N/A | Mixed |
| *IFNGR1* | +1.16 * | −0.12 ns | N/A | Mixed |

*Our cohort: qRT-PCR data. GSE51675: Agilent GPL6480 microarray, chronic antibody-mediated rejection (CAMR; n = 10) versus stable graft (n = 8). GSE145408: Affymetrix GPL13158 microarray, tacrolimus withdrawal (W; n = 18) versus maintenance (M; n = 18) — only six genes were queried in this dataset because the array probe set was not fully overlapping with the panel. All comparisons used Mann–Whitney U with Benjamini–Hochberg false-discovery-rate correction. Direction column summarises the consensus direction across cohorts; “Mixed” denotes datasets giving opposite signs without statistical significance. ***p < 0.001; **p < 0.01; *p < 0.05; ns, non-significant; N/A, gene not present on the array.*
